# Shock Index, Pediatric Age-Adjusted Predicts Morbidity and Mortality in Children Admitted to the Intensive Care Unit

**DOI:** 10.3389/fped.2021.727466

**Published:** 2021-09-28

**Authors:** Kuo-Chen Huang, Ying Yang, Chao-Jui Li, Fu-Jen Cheng, Ying-Hsien Huang, Po-Chun Chuang, I-Min Chiu

**Affiliations:** ^1^Department of Emergency Medicine, Kaohsiung Chang Gung Memorial Hospital, Kaohsiung, Taiwan; ^2^Department of Pediatrics, Kaohsiung Chang Gung Memorial Hospital, Kaohsiung, Taiwan

**Keywords:** pediatric, SIPA, shock index, mortality, emergency department, intensive care unit

## Abstract

**Background:** The shock index, pediatric age-adjusted (SIPA), defined as the maximum normal heart rate divided by the minimum normal systolic blood pressure by age, can help predict the risk of morbidity and mortality after pediatric trauma. This study investigated whether the SIPA can be used as an early index of prognosis for non-traumatic children visiting the pediatric emergency department (ED) and were directly admitted to the intensive care unit (ICU). We hypothesized that an increase in SIPA values in the first 24 h of ICU admission would correlate with mortality and adverse outcomes.

**Methods:** This multicenter retrospective study enrolled non-traumatic patients aged 1–17 years who presented to the pediatric ED and were directly admitted to the ICU from January 1, 2016, to December 31, 2018, in Taiwan. The SIPA value was calculated at the time of arrival at the ED and 24 h after ICU admission. Cutoffs included SIPA values >1.2 (patient age: 1–6), >1.0 (patient age: 7–12), and >0.9 (patient age: 12–17). The utility of the SIPA and the trends in the SIPA during the first 24 h of ICU admission were analyzed to predict outcomes.

**Results:** In total, 1,732 patients were included. Of these, 1,050 (60.6%) were under 6 years old, and the median Pediatric Risk of Mortality score was 7 (5–10). In total, 4.7% of the patients died, 12.9% received mechanical ventilator (MV) support, and 11.1% received inotropic support. The SIPA value at 24 h after admission was associated with increased mortality [odds ratio (OR): 4.366, 95% confidence interval (CI): 2.392–7.969, *p* < 0.001], MV support (OR: 1.826, 95% CI: 1.322–2.521, *p* < 0.001), inotropic support (OR: 2.306, 95% CI: 1.599–3.326, *p* < 0.001), and a long hospital length of stay (HLOS) (2.903 days, 95% CI: 1.734–4.271, *p* < 0.001). Persistent abnormal SIPA value was associated with increased mortality (OR: 2.799, 95% CI: 1.566–5.001, *p* = 0.001), MV support (OR: 1.457, 95% CI: 1.015–2.092, *p* = 0.041), inotropic support (OR: 1.875, 95% CI: 1.287–2.833, *p* = 0.001), and a long HLOS (3.2 days, 95% CI: 1.9–4.6, *p* < 0.001). Patients with abnormal to normal SIPA values were associated with decreased mortality (OR: 0.258, 95% CI: 0.106–0.627, *p* = 0.003), while patients with normal to abnormal SIPA values were associated with increased mortality (OR: 3.055, 95% CI: 1.472–5.930, *p* = 0.002).

**Conclusions:** In non-traumatic children admitted to the ICU from the ED, increased SIPA values at 24 h after ICU admission predicted high mortality and bad outcomes. Monitoring the trends in the SIPA could help with prognostication and optimize early management.

## Introduction

The shock index (SI), defined as the ratio of heart rate (HR) to systolic blood pressure (SBP), is widely used as a predictor in patients presenting with trauma or acute hemorrhage (1, 2). Studies have shown the advantages of SI and adjusted SI values over conventional vital sign measurements in detecting occult hypovolemic shock and predicting mortality (3–6). The SI has been used to predict mortality in patients with medical conditions (7), such as sepsis (8, 9), pulmonary embolism (10), and community-acquired pneumonia (11). Furthermore, changes in SI values over time predict mortality in non-traumatic critically ill patients (12, 13). In the pediatric population, the SI can also be useful in predicting mortality in children with septic shock (14, 15).

There is an age-specific normal limit of vital signs in pediatric patients. The normal range of SI values may differ across different age groups. Hence, the SI, pediatric age-adjusted (SIPA), defined as the maximum HR divided by minimum SBP from an age-specific normal limit, was developed (Table 1) (16–19). Shannon N. Acker et al. demonstrated that the SIPA can more accurately identify severely injured children following blunt trauma than the SI unadjusted for age (18, 20). The SIPA is better at predicting outcomes, such as the need for emergency surgery, endotracheal intubation, early blood transfusion, intensive care unit (ICU), and long hospital length of stay (HLOS) and mortality (17, 21–23). Recent studies further illustrated the use of following the trends in the SIPA with regard to important predictors of outcomes in the pediatric population experiencing trauma (24–26).

**Table 1:** Vital signs by age with the calculated SIPA cutoff value.

**Age**	**HR**	**SBP**	**SIPA cutoff value**
1–3 years	70–110	90–110	1.2
4–6 years	65–110	90–110	1.2
7–12 years	60–100	100–120	1.0
>12 years	55–90	100–135	0.9

*SIPA, shock index, pediatric age-adjusted; HR, heart rate; SBP, systolic blood pressure*.

There is a paucity of data on this subject in non-traumatic pediatric patients. One study conducted in 2020 indicated that febrile children with elevated SIPA values on the initial emergency department (ED) visit were associated with ICU admission following an unscheduled ED revisit within 72 h (27). Considering that the conclusions of most studies on SIPA were confined to trauma patients, this study attempted to expand the use of this tool to include additional pediatric populations.

To investigate whether the SIPA can be used as an early index of prognosis for non-traumatic children visiting the pediatric ED and were directly admitted to the ICU, this study compared the association of the SI and SIPA with clinical characteristics, mortality, and adverse outcomes. Moreover, we analyzed the utility of trends in the SIPA in predicting mortality and adverse outcomes in the first 24 h of ICU admission. We hypothesized that an increase in SIPA values in the first 24 h of ICU admission would correlate with adverse outcomes in non-traumatic children.

## Materials and Methods

This retrospective observational study was conducted at the EDs of three medical institutes (the Linkou, Chiayi, and Kaohsiung branches) located in northern and southern Taiwan. There were 35,000, 11,000, and 30,000 annual pediatric ED visits, respectively. The study was approved by the Institutional Review Board of the Chang Gung Medical Foundation (date of approval: May 15, 2021, number: 202100692B0). Patient and physician records and information were collected from the research database in the studied medical foundation and anonymized prior to the analysis.

**Figure 1:**
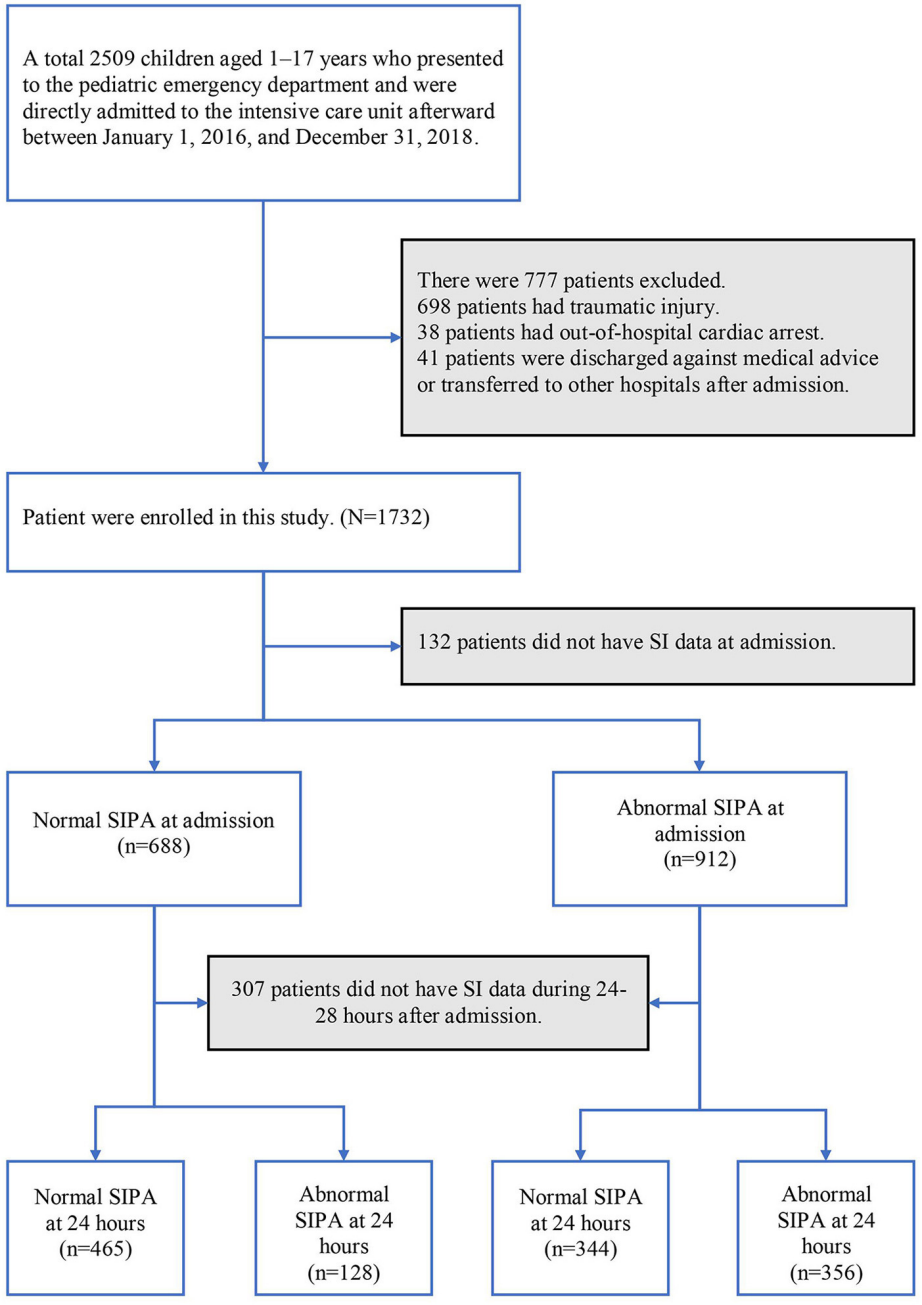
Patient inclusion flowchart. SI, shock index; SIPA, shock index, pediatric age-adjusted.

We enrolled children aged 1–17 years who presented to the pediatric ED and were directly admitted to the ICU between January 1, 2016, and December 31, 2018. Patients aged <1 year were excluded because the normal range of vital signs varied drastically by month and the normal range of SIPA values in this population was not clearly categorized in previous studies. Patients who experienced traumatic injury, who had out-of-hospital cardiac arrest, were discharged against medical advice, or were transferred to other hospitals after admission were also excluded. Figure 1 shows a patient inclusion flowchart.

We calculated the SI and SIPA values from medical records documented in the research database for the included patients at the time of ED arrival and 24 h after admission. These values were then categorized as either abnormal (i.e., above the normal SI range or normal SIPA value for age range) or normal based on the normal range of vital signs in different age groups that were adopted in recent studies (17, 25, 27). We also calculated delta SI by subtracting the SI value at 24 h after admission from the SI value at admission. We then compared their association with patient severity and clinical outcomes. Information on demographic variables including age, sex, and patients' clinical characteristics was collected to obtain the severity index and the Pediatric Risk of Mortality (PRISM) score (28) in order to determine the level of correlation of the SI and SIPA with severity.

The primary outcome was mortality at ICU admission. The secondary outcomes included mechanical ventilator (MV) support, inotropic agent support, and the HLOS. To further investigate the utility of using the SIPA as a tool in clinical monitoring, we used the trends of the SIPA value at admission and 24 h later to correlate with clinical outcomes. In this step of the analysis, four different trends of the SIPA were analyzed: normal to normal, normal to abnormal, abnormal to normal, and abnormal to abnormal SIPA values during the first 24 h.

The Mann-Whitney *U* test for continuous variables and chi-square analysis for binomial variables were performed to determine the clinical characteristics and outcomes that were correlated with the SI and SIPA. Logistic regressions assessing the association of clinical outcomes with the SIPA value and the trends in the SIPA during the first 24 h were performed after adjusting for confounding factors. Statistical significance was defined as a two-sided *p*-value < 0.05. All statistical analyses were conducted using the IBM SPSS Statistics for Mac (version 26).

## Results

During the study period, 1,732 patients were included in the study. Of these, 1,050 (60.6%) were under 6 years old, 1,010 (58.3%) were male, and the median PRISM score was 7 (5–10). In total, 4.7% of the patients died, 12.9% received MV support, and 11.1% received inotropic support (Table 2).

**Table 2:** Demographics and clinical characteristics of the included patients.

**Variables**	**Median (IQR)/*N* (%)**
Total patients	1,732
**Hospitals**	
Northern	641 (37.0%)
Middle-west	194 (11.2%)
Southern	897 (51.8%)
Age	4 (1–12)
1–3	840 (48.5%)
4–6	210 (12.1%)
7–12	296 (17.1%)
13–17	386 (22.3%)
**Sex**	
Male	1,010 (58.3%)
Female	722 (41.7%)
PRISM III	7 (5–10)
**Shock index**	
At admission	1.15 (0.92–1.44)
At 24 h	1.02 (0.80–1.27)
Delta SI	−0.12 (−0.31–0.07)
**Outcome**	
Mortality	82 (4.7%)
Ventilator support	224 (12.9%)
Inotropic support	192 (11.1%)
HLOS, day	7 (4-11)

*IQR, interquartile range; SI, shock index; HLOS, hospital length of stay; PRISM, Pediatric Risk of Mortality*.

The association of SI and SIPA with clinical appearance and outcomes is shown in Table 3. At admission, 1,230 (76.9%) patients were categorized as having abnormal SI values, while 912 (57.0%) patients were considered to have abnormal SIPA values. The mean patient age was significantly higher in both the normal SI [13 (9–16) vs. 3 (1–9), *p* < 0.001] and SIPA [7 (2–14) vs. 3 (1–11), *p* < 0.001) groups than in the abnormal group. There was a statistically significant difference in PRISM scores between the normal and abnormal SIPA groups [6 (5–10) vs. 9 (5–10), *p* = 0.039], while there was no significant difference between the normal and abnormal SI groups [8 (5–10) vs. 7 (5–10), *p* = 0.264]. Regarding clinical outcomes, neither abnormal SI nor abnormal SIPA values at admission were significantly correlated with in-hospital mortality, MV support, and inotropic support. An abnormal SIPA value was associated with increased HLOS [7 (5–12) vs. 6 (4–10), *p* = 0.003].

**Table 3:** Correlation of SIPA at admission and 24 hours later with patient outcome.

		**SI**			**SIPA**		
		**Normal** **median (IQR)/*N*%**	**Abnormal** **median (IQR)/*N*%**	***P*** **-value**	**Normal** **median (IQR)/*N*%**	**Abnormal** **median (IQR)/*N*%**	***P*** **-value**
At admission	Total	370 (23.1%)	1,230 (76.9%)		688 (43.0%)	912 (57.0%)	
	Age	13 (9–16)	3 (1–9)	<0.001	7 (2–14)	3 (1–11)	<0.001
	PRISM	8 (5–10)	7 (5–10)	0.264	6 (5–10)	9 (5–10)	0.039
	Mortality	16 (4.3%)	56 (4.5%)	1.000	30 (4.4%)	50 (5.5%)	0.355
	MV support	52 (14.1%)	143 (11.6%)	0.205	83 (12.2%)	117 (12.9%)	0.760
	Inotropic support	41 (11.1%)	132 (10.7%)	0.849	69 (10.2%)	109 (12.0%)	0.261
	HLOS, day	6 (4–10)	7 (4–11)	0.147	6 (4–10)	7 (5–12)	0.003
At 24 h	Total	500 (35.9%)	893 (64.1%)		844 (60.6%)	549 (39.4%)	
	Age	13 (7–15)	2 (1–6)	<0.001	7 (3–14)	2 (1–9)	<0.001
	PRISM	8 (5–10)	7 (5–10)	0.139	8 (5–10)	8 (5–10)	0.530
	Mortality	14 (2.8%)	46 (5.2%)	0.039	19 (2.3%)	39 (7.1%)	<0.001
	MV support	62 (12.4%)	142 (15.9%)	0.082	99 (11.9%)	98 (18.1%)	0.002
	Inotropic support	51 (10.2%)	115 (12.9%)	0.144	78 (9.4%)	84 (15.5%)	0.001
	HLOS, day	7 (4–11)	7 (5–12)	0.147	7 (4–11)	8 (5–13)	0.001

*IQR, interquartile range; SI, shock index; HLOS, hospital length of stay; PRISM, Pediatric Risk of Mortality; SIPA, shock index, pediatric age-adjusted; MV, mechanical ventilator*.

Considering the vital sign index at 24 h after admission, the ratio of abnormal SIPA values dropped to 39.4%, while abnormal SI values accounted for 64.1% of the total patients. The mean patient age was significantly higher in both the normal SI [13 (7–15) vs. 2 (1–6), *p* < 0.001] and SIPA [7 (3–14) vs. 2 (1–9), *p* < 0.001] groups than in the abnormal group. Abnormal SIPA values at 24 h were associated with all measured outcomes, including high mortality (7.1 vs. 2.3%, *p* < 0.001), MV support (18.1 vs. 11.9%, *p* = 0.002), inotropic support (15.5 vs. 9.4%, *p* = 0.001), and a long HLOS [8 (5–13) vs. 7 (4–11), *p* = 0.001). On the contrary, abnormal SI values were significantly associated with high mortality (5.2 vs. 2.8%, *p* = 0.039), but not with other secondary outcomes.

We performed a regression analysis on the correlation of SIPA values at admission, 24 h after admission, and delta SI with the measured outcomes. After adjusting for age, sex, and patient severity, SIPA values at admission were associated with increased HLOS [1.282 days, 95% confidence interval (CI): 0.240–2.324, *p* = 0.016], but not with a high rate of mortality or other secondary outcomes. In contrast, SIPA values at 24 h after admission were associated with increased mortality [odds ratio (OR): 4.366, 95% CI: 2.392–7.969, *p* < 0.001], MV support (OR: 1.826, 95% CI: 1.322–2.521, *p* < 0.001), inotropic support (OR: 2.306, 95% CI: 1.599–3.326, *p* < 0.001), and HLOS (2.903 days, 95% CI: 1.734–4.271, *p* < 0.001). Furthermore, delta SI was associated with increased mortality (OR: 8.158, 95% CI: 3.838–17.340, *p* < 0.001), MV support (OR: 3.309, 95% CI: 1.638–6.683, *p* < 0.001), inotropic support (OR: 4.233, 95% CI: 2.454–7.303, *p* < 0.001), but not with HLOS.

**Figure 2:**
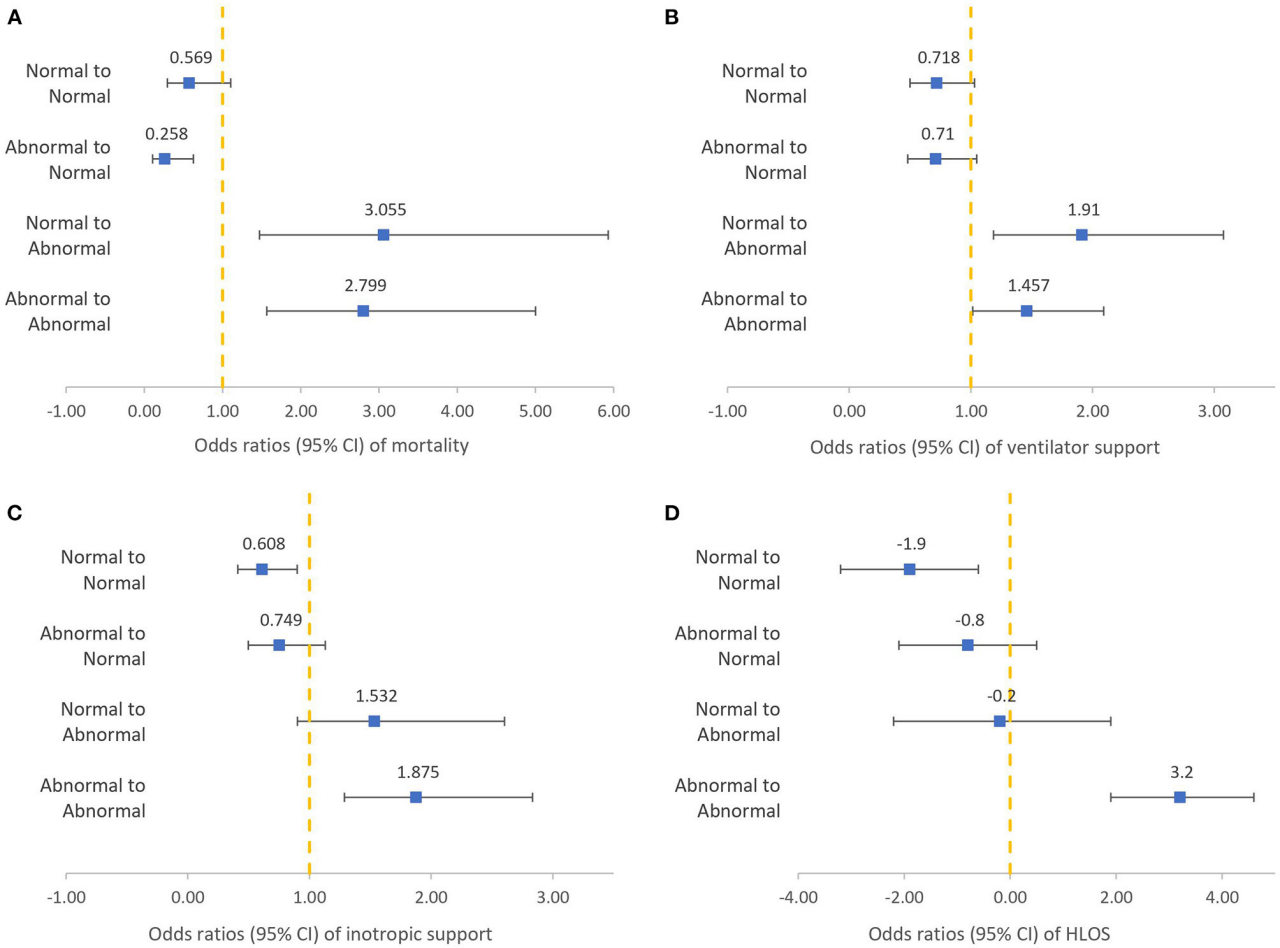
Logistic regression of different shock index, pediatric age-adjusted trends to clinical outcomes after adjusting for confounding factors: mortality **(A)**, ventilator support **(B)**, inotropic support **(C)**, HLOS **(D)**.

The results of the SIPA trends in terms of patient outcomes are shown in Figure 2. We found that patients with persistent abnormal SIPA values during the first 24 h were significantly associated with adverse outcomes, including increased mortality (OR: 2.799, 95% CI: 1.566–5.001, *p* = 0.001), MV support (OR: 1.457, 95% CI: 1.015–2.092, *p* = 0.041), inotropic support (OR: 1.875, 95% CI: 1.287–2.833, *p* = 0.001), and HLOS (3.2 days, 95% CI: 1.9–4.6, *p* < 0.001). In contrast, patients with normal SIPA values in the first 24 h were associated with a decreased HLOS (−1.901 days, 95% CI: −3.181 −0.632, *p* = 0.002). Patients with abnormal to normal SIPA values were associated with decreased mortality (OR: 0.258, 95% CI: 0.106–0.627, *p* = 0.003). In contrast, patients with normal to abnormal SIPA values were associated with increased mortality (OR: 3.055, 95% CI: 1.472–5.930, *p* = 0.002) and increased MV support (OR: 1.910, 95% CI: 1.186–3.076, *p* = 0.008).

## Discussion

Studies have proven the SIPA to be a useful tool for predicting the risk of morbidity and mortality in the pediatric population experiencing trauma (17, 18, 24). This study further demonstrates the utility of elevated SIPA values and the trends in the SIPA for the first 24 h in predicting outcomes among pediatric non-traumatic patients. As shown in Table 3, abnormal SIPA values at 24 h accurately identified children at a high risk of adverse outcomes, including high mortality, MV support, inotropic support, and a long HLOS. Although both elevated SI and elevated SIPA values at 24 h after admission appear to be associated with high mortality, making pediatric adjustment unnecessary, elevated SIPA values at 24 h after admission correlated with each of the three outcomes measured. The same results were obtained after adjusting for confounding factors (Table 4). It should be noted that there were 19 children with normal SIPA values at 24 h who died. Among these, eight were diagnosed with cancer, four had congenital disorders, six had central nervous system anomalies, and one had heart failure after acute myocarditis. The SIPA should be used with caution in patients with severe underlying diseases.

**Table 4:** Logistic regression analysis of the association of SIPA to clinical outcomes after adjusting confounding factors.

**SIPA**		**Odds ratio (95% CI)**	***P*** **-value**
At admission	Mortality	1.098 (0.670–1.798)	0.711
	Ventilator support	1.061 (0.775–1.452)	0.712
	Inotropic support	1.257 (0.902–1.753)	0.177
	HLOS	1.282 (0.240–2.324)	0.016
At 24 h	Mortality	4.366 (2.392–7.969)	<0.001
	Ventilator support	1.826 (1.322–2.521)	<0.001
	Inotropic support	2.306 (1.599–3.326)	<0.001
	HLOS	2.903 (1.734–4.271)	<0.001
Delta SI	Mortality	8.158 (3.838–17.340)	<0.001
	Ventilator support	3.309 (1.638–6.683)	<0.001
	Inotropic support	4.233 (2.454–7.303)	<0.001
	HLOS	0.900 (−0.725–2.525)	0.277

*CI, confidence interval; SI, shock index; HLOS, hospital length of stay; SIPA, shock index, pediatric age-adjusted*.

Moreover, there was a significant difference in PRISM scores between the normal and abnormal SIPA groups at admission. This finding indicates that the SIPA more accurately identifies a patient's severity than the SI unadjusted for age. Interestingly, elevated SIPA values at admission were only associated with an increased HLOS, whereas elevated SI values at admission were not associated with any measured outcomes. This discrepancy could be a consequence of early intervention and management, which may alter patient outcomes. Children presenting to the ED with initially unstable conditions receive aggressive resuscitation and a high level of care; thus, children with abnormal SIPA values at admission can avoid adverse outcomes. Additionally, the average age of the patients was high in both the normal SI and SIPA groups. This may be related to the limited physiological ability of compensation in response to disease progression in young children.

Nordin et al. demonstrated that the change in SIPA values in the prehospital period predicts the need for transfusions, ICU admission, and mortality in pediatric trauma patients (25). A study conducted in 2018 also indicated that a normal SIPA value at the time of arrival to the hospital, which becomes elevated in the first 48 h of admission, correlates strongly with adverse outcomes in pediatric blunt trauma patients (24). This study provides additional evidence for SIPA trends in predicting outcomes in children. Alterations in SIPA values during the first 24 h were associated with adverse outcomes, as shown in Figure 2. When we studied children with persistently elevated SIPA values, either at admission or at 24 h after admission, we observed an increased risk of mortality, MV support, inotropic support, and HLOS. Additionally, progression from a normal SIPA value to an abnormal SIPA value is also associated with increased mortality and the need for MV. Conversely, improvement of SIPA values within 24 h of ICU admission was associated with decreased mortality in children with high SIPA values at admission. This result suggests that monitoring changes in SIPA values, especially persistently elevated SIPA values within 24 h of ICU admission, could provide information to help predict adverse outcomes. Such information may alert healthcare providers to the risk of poor prognosis among children admitted to the ICU. It also offers healthcare providers a clue to communicate their therapeutic plan to family members and to explain indications for invasive procedures, such as central venous catheterization.

Delta SI can also be used to predict adverse outcomes. As shown in Table 4, delta SI was strongly associated with mortality, MV support, and inotropic agent use. However, the normal range of SIPA values may differ across different age groups due to age-specific normal limits of vital signs in pediatric patients. Therefore, the dichotomization of SIPA values as normal or abnormal may be a concise and explicit way for clinical application.

The main advantage of SIPA is that the values can be rapidly calculated at the bedside. The required parameters are easily obtained from routine vital signs. The SIPA should be integrated into electronic medical records and automatically calculated in real time according to HR and SBP. The utility of SIPA monitoring may allow early recognition of children with poor outcomes, and be an indicator for early implementation of aggressive treatment. Further research is needed to evaluate the improvement in early management among children admitted to the ICU after SIPA monitoring.

This study has several limitations. First, it had a retrospective study design; thus, all data collection depended on the information documented in the medical records. Second, this study was conducted at the ED of three medical institutions. We did not analyze interfacility differences in practice, such as ICU admission criteria or indications for inotropic agent administration. However, we believe that this would have a minimal effect on our results, as all the studied hospitals have similar systems. Third, the sample population was limited to children aged 1–17 years. The applicability of these findings to patients younger than 1 year is unknown and should be the focus of future work. Finally, we used non-invasive SBP measurements for SI calculation in all patients included. Accurate measurement of vital signs in young children is challenging. The measurement of vital signs may differ among nursing staff who are unaccustomed to caring for children. Nevertheless, the effect should be minimal, as this would have affected both the calculated SI and SIPA values equally. The usefulness of the SIPA should be tested in large prospective cohorts of non-traumatic children. Additional resuscitation metrics (that is, the serum lactate level, central venous oxygen saturation, or serum base deficit) may be incorporated for a precise prediction of prognosis.

In conclusion, elevated SIPA values at 24 h after ICU admission predicts high mortality and bad outcomes. Monitoring the trends in the SIPA could provide information for predicting adverse outcomes in non-traumatic children visiting the pediatric ED, directly admitted to the ICU. It is practicable to add the SIPA to HR and SBP, allowing for the prognostication and optimization of early management.

## Data Availability Statement

The raw data supporting the conclusions of this article will be made available by the authors, without undue reservation.

## Ethics Statement

The study was approved by the Institutional Review Board of the Chang Gung Medical Foundation (date of approval: May 15, 2021, number: 202100692B0). Patient and physician records and information were collected from the research database in the studied medical foundation and anonymized prior to the analysis.

## Author Contributions

I-MC and Y-HH contributed to the conception and design of the study. P-CC performed the statistical analysis and data interpretation. K-CH, YY, C-JL, and F-JC contributed to drafting the article and reviewing the literature. All authors contributed to the manuscript revision and approved the submitted version.

## Conflict of Interest

The authors declare that the research was conducted in the absence of any commercial or financial relationships that could be construed as a potential conflict of interest.

## Publisher's Note

All claims expressed in this article are solely those of the authors and do not necessarily represent those of their affiliated organizations, or those of the publisher, the editors and the reviewers. Any product that may be evaluated in this article, or claim that may be made by its manufacturer, is not guaranteed or endorsed by the publisher.
