# Corrigendum: Shock Index, Pediatric Age-Adjusted Predicts Morbidity and Mortality in Children Admitted to the Intensive Care Unit

**DOI:** 10.3389/fped.2021.788361

**Published:** 2021-11-17

**Authors:** Kuo-Chen Huang, Ying Yang, Chao-Jui Li, Fu-Jen Cheng, Ying-Hsien Huang, Po-Chun Chuang, I-Min Chiu

**Affiliations:** ^1^Department of Emergency Medicine, Kaohsiung Chang Gung Memorial Hospital, Kaohsiung, Taiwan; ^2^Department of Pediatrics, Kaohsiung Chang Gung Memorial Hospital, Kaohsiung, Taiwan

**Keywords:** pediatric, SIPA, shock index, mortality, emergency department, intensive care unit

In the original article, the Author Contributions statement was missing the phrase “K-CH and YY contributed equally to this article.” The corrected Author Contributions statement appears below.

“I-MC and Y-HH contributed to the conception and design of the study. P-CC performed the statistical analysis and data interpretation. K-CH, YY, C-JL, and F-JC contributed to drafting the article and reviewing the literature. All authors contributed to the manuscript revision and approved the submitted version.”

The authors apologize for this error and state that this does not change the scientific conclusions of the article in any way. The original article has been updated.

## Publisher's Note

All claims expressed in this article are solely those of the authors and do not necessarily represent those of their affiliated organizations, or those of the publisher, the editors and the reviewers. Any product that may be evaluated in this article, or claim that may be made by its manufacturer, is not guaranteed or endorsed by the publisher.

