# Corrigendum: Shock Index, Pediatric Age-Adjusted Predicts Morbidity and Mortality in Children Admitted to the Intensive Care Unit

**DOI:** 10.3389/fped.2021.827191

**Published:** 2021-12-21

**Authors:** Kuo-Chen Huang, Ying Yang, Chao-Jui Li, Fu-Jen Cheng, Ying-Hsien Huang, Po-Chun Chuang, I-Min Chiu

**Affiliations:** ^1^Department of Emergency Medicine, Kaohsiung Chang Gung Memorial Hospital, Kaohsiung, Taiwan; ^2^Department of Pediatrics, Kaohsiung Chang Gung Memorial Hospital, Kaohsiung, Taiwan

**Keywords:** pediatric, SIPA, shock index, mortality, emergency department, intensive care unit

In the original article, there was an error. The date of approval of the study was incorrectly listed as “May 15, 2020.” The study was approved by the Institutional Review Board of the Chang Gung Medical Foundation on May 15, 2021.

A correction has been made to **Materials and Methods**, Paragraph 1:

“This retrospective observational study was conducted at the EDs of three medical institutes (the Linkou, Chiayi, and Kaohsiung branches) located in northern and southern Taiwan. There were 35,000, 11,000, and 30,000 annual pediatric ED visits, respectively. The study was approved by the Institutional Review Board of the Chang Gung Medical Foundation (date of approval: May 15, 2021, number: 202100692B0). Patient and physician records and information were collected from the research database in the studied medical foundation and anonymized prior to the analysis.”

A correction has been made to **Ethics Statement**:

“The study was approved by the Institutional Review Board of the Chang Gung Medical Foundation (date of approval: May 15, 2021, number: 202100692B0). Patient and physician records and information were collected from the research database in the studied medical foundation and anonymized prior to the analysis.”

The authors apologize for this error and state that this does not change the scientific conclusions of the article in any way. The original article has been updated.

## Publisher's Note

All claims expressed in this article are solely those of the authors and do not necessarily represent those of their affiliated organizations, or those of the publisher, the editors and the reviewers. Any product that may be evaluated in this article, or claim that may be made by its manufacturer, is not guaranteed or endorsed by the publisher.

